# Automated algorithm for medical data structuring, and segmentation using artificial intelligence within secured environment for dataset creation

**DOI:** 10.1016/j.ejro.2024.100582

**Published:** 2024-06-27

**Authors:** Varatharajan Nainamalai, Hemin Ali Qair, Egidijus Pelanis, Håvard Bjørke Jenssen, Åsmund Avdem Fretland, Bjørn Edwin, Ole Jakob Elle, Ilangko Balasingham

**Affiliations:** a The Intervention Centre, Rikshospitalet, Oslo University Hospital, Oslo, Norway; b Institute of Clinical Medicine, University of Oslo, Oslo, Norway; c Department of Radiology and Nuclear Medicine, Oslo University Hospital, Oslo, Norway; d Department of Hepato-Pancreatic-Biliary surgery, Oslo University Hospital, Oslo, Norway; e Department of Informatics, University of Oslo, Oslo, Norway; f Department of electronic systems (IES), Norwegian University of Science and Technology, Trondheim, Norway

**Keywords:** Electronic health records, Artificial intelligence, Structured data, Segmentation, Ground truth creation

## Abstract

**Objective:**

Routinely collected electronic health records using artificial intelligence (AI)-based systems bring out enormous benefits for patients, healthcare centers, and its industries. Artificial intelligence models can be used to structure a wide variety of unstructured data.

**Methods:**

We present a semi-automatic workflow for medical dataset management, including data structuring, research extraction, AI-ground truth creation, and updates. The algorithm creates directories based on keywords in new file names.

**Results:**

Our work focuses on organizing computed tomography (CT), magnetic resonance (MR) images, patient clinical data, and segmented annotations. In addition, an AI model is used to generate different initial labels that can be edited manually to create ground truth labels. The manually verified ground truth labels are later included in the structured dataset using an automated algorithm for future research.

**Conclusion:**

This is a workflow with an AI model trained on local hospital medical data with output based/adapted to the users and their preferences. The automated algorithms and AI model could be implemented inside a secondary secure environment in the hospital to produce inferences.

## 1 Introduction

Analyzing unstructured Electronic Health Records (EHRs) using artificial intelligence (AI) - based systems brings out a wide variety of benefits [1], [2], [3], [4], [5]. The primary advantages of implementing EHR are to bring quality in health care, reduction of medication errors, effective communication between clinicians, help to control costs, lead to better treatment, easy administrative purposes, etc. [6], [7], [8], [9]. The secondary utilization and analysis of healthcare data using AI-based algorithms avail a wide variety of benefits [10], [11]. Though the analysis of EHR data provides huge benefits, inherently it has numerous challenges while processing health data, such as data harmonization, integration and storage, inflexibility of EHRs, data privacy and security, fairness and bias, data quality and variability, complexity, noisiness, etc. [9], [12]. Currently, there are not many solutions for automated data structuring and cleaning that can ease utilizing data for research purposes [12].

A significant amount of EHR data lies in an unstructured form which means the data is not managed or arranged and searched through a relational database management system. The unstructured form of data includes clinical notes, discharge summaries, any documents generated on behalf of patients, medical images such as ultrasound, radiographs, MR images, CT images, angiography, histopathology images, etc [9], [13]. Unstructured data is as important as structured data, and it is estimated that around 80 % of the unstructured data is still not utilized. More benefits can be obtained when structured and unstructured data are combined for clinical analysis [14], [15], [16].

Handling medical data is one of the big data problems since the rapid growth of stored digital clinical data every year. There are a few automatic data extraction methods that exist. A customized extraction program, an automated data extraction model, was evaluated for its efficiency and shows extracted data accurately of 97.5 % [17]. A series of MR images were first structured and then many computational analyses were performed on the structured data [18], [19]. XNAT, an open-source imaging informatics platform, follows a three-level architecture for structuring and accessing the data [18], [20]. Recently, a deep learning (DL) model was developed and applied to cross documents of patient data such as pathology reports, radiology reports, and progress notes from cancer registry data for structuring, and it has shown promising performance with high accuracy [21].

The Brain Imaging Data Structure (BIDS) was developed to structure, store, organize, and archive neuroimaging MR data [22]. Afterward, BIDS has been extended to many other forms of patient data including magnetoencephalography [23], electroencephalography [24], electro- physiology [25], [26], [27], positron emission tomography [28], quantitative MR images [29], [30], microscopy imaging data [31], arterial spin labeling [32], multicentre data structure [33], genetic BIDS [34], etc. A few researchers have simplified the handling of the BIDS, using containers [35], NeuroPycon (an open-source Python-based toolkit for advanced multi-threaded processing of multi-modal brain data, including fMRI, MEG, and EEG analysis) [36], FlywheelTools [37], Brain Predictability toolbox (a Python-based machine learning library used for neuroimaging data analysis) [38], CuBIDS (Curation of BIDS) [39], and BIDScoin (a Graphical User Interface Python application suite that converts (“coins”) source-level (raw) neuroimaging data) [40] to extend BIDS functionality to help BIDS users to process data efficiently.

The Medical Imaging Data Structure (MIDS) was introduced to structure the data for the other anatomical regions and other types of imaging modalities [41]. OpenNeuro package was introduced to analyze and share neuroimaging data based on the BIDS. The structured dataset also needs continuous updates from a research project or when there is a new patient or additional data for an existing patient. If not handled and structured following any of the above-mentioned ways, adding newly generated ground truth labels leads to the unstructured data problem. Data extraction for research for researchers from a given dataset is a difficult task due to the unstructured data in combination with data security and protection.

In this paper, we introduce a semi-automatic workflow for structuring and continuously up-dating datasets using BIDS inspired algorithm to reduce the workload of manually handling a dataset. The proposed semi-automatic workflow serves the following purposes: Data structuring, data extraction, AI training and inferencing, and semi-automatic updating of the dataset. Our contribution is that the idea of automatically structuring the data, anonymization of data, and automatically extraction of data using this proposed algorithm. An important key point of this workflow is that it is independent of any data modalities.

The structured directories are dependent on the keywords in the file names and the path to the directory where the file is located. We restrict our implementation to structuring CT images, different MR image sequences, patient data, and segmentation in a form of a label map for each individual structure. In addition, a AI-based model is used and trained to generate inferences of different class labels that can be used as ground truth labels after being manually verified. The manually verified ground truth labels are added to the main structured dataset using a semi-automatic algorithm to serve as the final ground truth labels for future research. Most important, as data is structured in the same way inside and outside the database-trained AI models can easily be transferred and run for inference on sensitive data. All these works could potentially be done inside a secondary secure environment in the hospital.

The workflow and the proposed algorithms will be explained in Section 2. The test case study on CT images, a dataset that is to be structured, the ground truth creation process, results, and discussions will be discussed in further sections.

## 2 Methods

### 2.1 Ethical approval

For the development and testing of the proposed workflow, a dataset was created with data from originating from the Oslo laparoscopic versus open liver resection for colorectal liver metastases clinical trial study (OSLO-COMET).

The study was performed in accordance with the ethical standards of the institutional and/or national research committee and with the 1964 Helsinki Declaration and its later amendments or comparable ethical standards. The project will comply with legislation on research ethics and data protection by following the policies given by Oslo University Hospital (ClinicalTrials.gov: NCT01516710).

The use of data in the OSLO-COMET trial has been approved by the Norwegian Regional Ethics Committee (REK Helse Sør-Øst nr. 2011/1285). The written informed consent has been obtained from all patients.

### 2.2 Anonymization and data conversion

The de-identification and anonymization of patient data and images are essential parts of data utilization for many retrospective and prospective studies. Throughout this article, de- identification represents that the structured dataset does not possess any information which can directly identify a patient. Although there exists a key to link back a patient that is always kept in the hospital-secured system. Anonymization means that the final structured dataset does not possess any information which can identify or relate to a patient. The legal regulations of the Health Insurance Portability and Accountability Act (HIPAA) in the United States and the General Data Protection Regulation (GDPR) in the European Union impose strict rules regarding the handling of personal data. We and the proposed workflow follow those imposed rules to de-identify and anonymize the data.

**Fig. 1:**
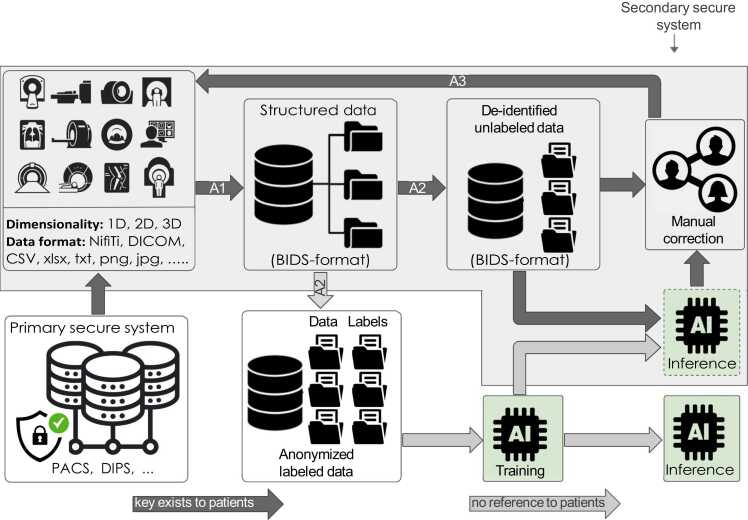
Workflow representation of the proposed automated dataset/database creation of secured hospital data for artificial intelligence study.

Initially, the de-identification is performed in the primary secure hospital system. Secondary de-identification and anonymization are performed in the secondary secure environment. If de-identified, the keys are used to add ground truth labels generated by an AI-based model after being manually verified by clinicians to the structured database in the secure secondary environment. We use Python programming to create algorithms for extracting, anonymizing, and structuring the data. The inferences of new data that do not possess ground truths are manually validated, and added again to the Medical Data Structure (MDS) dataset. The proposed framework to structure, de-identify, anonymize, and semi-automatically annotate clinical and image data with the help of a DL-based model in a secure manner is shown in Fig. 1.

### 2.3 File naming format

Radiological images and clinical test results data files were copied from the secured primary hospital system to a secondary secured environment. All the collected images and data are saved in this input source directory to a structured directory with a specific name pattern, explained below. The destination directory does not depend on the file format, and it is created based on the file name. The dataset consists of a patient directory for each patient that has an anonymized patient record as a JSON (JavaScript Object Notation) file. Consider an example H001_CT001_ Porto_IM.nii.gz for a clear explanation.

• The MAIN is the level one, main structured destination, directory of the proposed work- flow.
• The H001 is one of the sub-directories in the MAIN directory which is a level two directory. The number of level two sub-directories in the MAIN is equal to the number of patients enrolled in the clinical trial or data to be structured. It can take a three-digit number, though it could be expanded if needed, followed by H. The relationship between the number followed by H and the patient’s personal identification number is kept in the hospital’s primary security system.
• The sub-directory H001 has two objects namely, H001.json for the patient’s de-identified clinical records and the level three sub-directory CT001.
• The directory CT001 is a level three directory. The three-digit number is followed by image modality (CT or MR) which relates to the chronological order of the image acquired. It also contains a level four directory with the name of its phase or sequence of the modality for 3D Neuroimaging Informatics Technology Initiative (NIfTI) compressed format images.
• The level four sub-directory possesses 3D NIfTI format images and its different ground truth segmentations. The IM denotes the image, and nii.gz represents the compressed NIfTI image format.

### 2.4 Structuring data

Algorithm 1 (provided in Supplementary), denoted as A1 in Fig. 1, is developed to read the data from the source directory in the secondary secured system. Algorithm A1 checks each file in the source directory whether the file is JSON or not. If the file is not a JSON file, the name will be split based on underscores excluding the file format. The first string is used to create the level two directory if it does not exist. The second string is used to create the third-level directory if it does not exist. If the fourth level directory exists by checking the third string, then files will be moved, otherwise, it is created and files are moved into it.

**Fig. 2:**
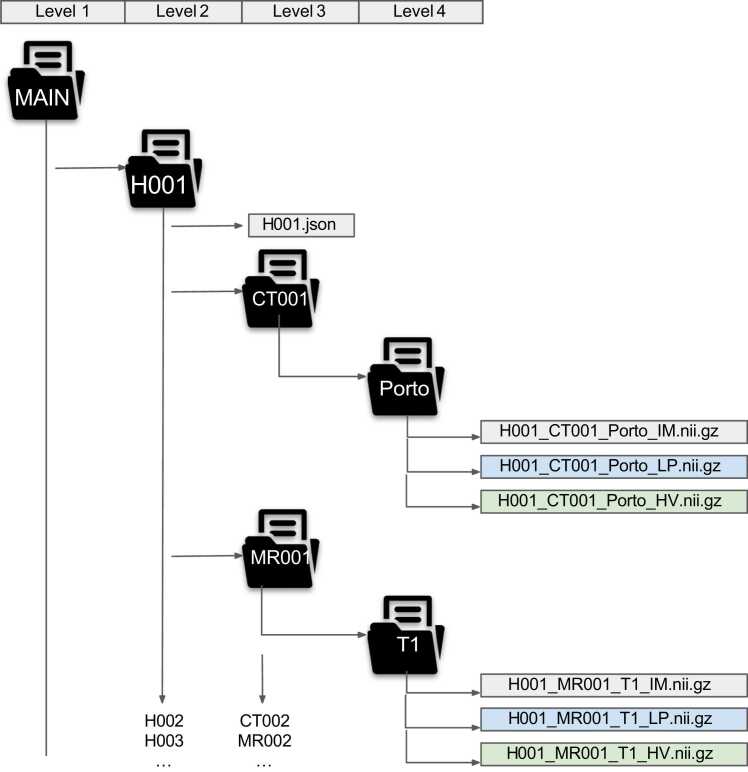
Overview of directory of the structured dataset. Porto-denotes the Portal venous phase of CT image. T1-denotes the T1 sequence of MR images. File names with IM, LP, and HV denotes the image, liver parenchyma, and hepatic vein respectively.

Algorithm A1 reads the comma-separated values (CSV) file in the source directory that contains clinical information such as measurements and records of clinical and lab data of all patients involved in a clinical trial. In the CSV file, the data in each row belongs to a patient. The Algorithm A1 read each row and saves every patient’s data in a single JSON which will be saved in the level two directory in the structured dataset. The single JSON is named as the de-identified patients’ id. as shown in Fig. 2.

### 2.5 Data extraction for annotation and research

Manually labeled image data or patient data or both can then be used to train an AI-based algorithm for a semi-automatic data annotation approach. However, in order to develop any AI-based models by utilizing data outside the secure environment, it is imperative that the data be fully anonymized. Hence, the data that is outside of the secure environment can never be linked back to the patients in the secure environment. Therefore, we propose Algorithm 2 (can be found as Supplementary Algorithm 2), denoted A2 in Fig. 1, which replaces the patients’ IDs with random numbers by an anonymization scheme. It has the following steps: First, a set of natural numbers **N** is created where **N** is equal to the number of patients’ data requested for a study. Then, the set **N** is randomly shuffled, denoted as **Ns**.

The data request for a study from a researcher is obtained in a specific CSV file format by specifying each patient image, and labels as a single row. All the requested data from each row of a CSV file is temporarily stored by the algorithm, called csvrows. Each element in the list csvrows is a list, and processed as follows: The first three elements are related to the level of sub-directories, and then it says the requirement of the image and its various label segmentations. Each image inside the fourth-level sub-directories is matched with the first, second, third, and fourth strings with an underscore with the file format for each element in the csvrows. If these strings are matched, then instead of H and three-digit numbers, replaced as ’Case’ and the first number of **Ns**, respectively. Once, the first number is used, that number is erased from **Ns**. Now, the new directory is created with a similar structure to MDS using a copy of A1, and copied data is moved to an external hard disk. The first numbered data is not the first data in the requested CSV file due to a random shuffle. In case, the same request CSV file is used twice consecutively, the order of the extracted data in both instances is different. The fully anonymized data will then be ready to be used by other researchers.

The algorithm A2 is also scripted to extract the unlabeled data inside the secondary secured environment. In this case, the patients’ IDs will be replaced with randomly shuffled natural numbers, and a log file that keeps those random numbers associated with the corresponding patients’ IDs will be created simultaneously. This log file is generated when there is a manual correction needed for the inferences by an AI-based model. This situation arises when there is new data.

### 2.6 Semi-automatic updating of manually verified ground truths

One of the objectives is to generate ground truth labels for anonymized data with minimal labeled input, employing an AI model. Once we have a trained AI model, it will then be applied to the de-identified unlabeled data to produce initial segmentation for ground truth creation. The key CSV file after the secondary de-identification and relates to the request CSV file at that instance will be kept in the secured primary hospital system. The manually corrected labels are renamed using the same CSV file. Algorithm 3 (can be found as Supplementary Algorithm 3), denoted as A3 in Fig. 1 is used to update the manually corrected labels to the source folder for the MDS structure. Also, the keyword IM is replaced with the new label name and then saved. The output folder for algorithm A3 is the source directory for A1. Finally, Algorithm A1 can structure the manually corrected labels from the source directory to the corresponding directories.

### 2.7 Segmentation metrics

The accuracy of AI-predicted labels are measured with the following metrics: The Dice similarity coefficient (DSC) is used to measure spatial overlap between ground truth and AI- predicted segmentations written as

(1) $$\mathrm{DSC}=\frac{2\left|\mathrm{GT}\cap \mathrm{Pred}\right|}{\left|\mathrm{GT}\right|\cup \left|\mathrm{Pred}\right|}$$

where GT and Pred are represents ground truth and AI predictions respectively. The Dice loss [42] can be written as Dice Loss=1*−*DSC.

Over segmentation measures the overlapping of false positives in the prediction and the complement of label class voxels in the manual segmentation over the union of labeled voxels in the manual segmentation and prediction defined by

(2) $$\mathrm{OS}=\frac{2\left|\overset{¯}{\mathrm{GT}}\cap \mathrm{Pred}\right|}{\left|\mathrm{GT}\right|\cup \left|\mathrm{Pred}\right|}$$

where GT complement of labels in the manual segmentation [43]. Under segmentation measures overlapping of the complement of label voxels in the prediction (Pred) and the manual segmentation over the union of labels in the manual segmentation and prediction defined by

(3) $$\mathrm{US}=\frac{2\left|\mathrm{GT}\cap \overset{¯}{\mathrm{Pred}}\right|}{\left|\mathrm{GT}\right|\cup \left|\mathrm{Pred}\right|}$$

## 3 Results

### 3.1 Dataset curation

Our dataset, OSLO-COMET, consists of CT images, MR images, and patent information obtained from various hospitals in Norway [44], [45], [46], [47]. The CT images were acquired using four manufacturers and 13 different models. The MR images were obtained using seven various models from two manufacturers and seven different models. The dataset has label segmentations complimented from various other studies, which were based on the same medical data.

At present, extraction of radiological and clinical data can be pulled from the primary secure hospital system into another secondary secure environment which can facilitate data manipulation and analysis. Medical images were de-identified in the primary secure hospital system picture archiving and communication system (PACS) itself during export. In this secondary environment, data can be stored as individual files and directories.

All medical images were in Digital Imaging and Communications in Medicine (DICOM) format and later converted into Neuroimaging Informatics Technology Initiative (NIfTI) compressed format. The compressed NIfTI format divides medical images into separate files for each medical volume and ensures the removal of potentially sensitive metadata present in the DICOM files. These NIfTI files are named in a systematic and consistent manner to keep a de-identified link to a specific patient, image modality, acquisition date, and volume acquisition information (protocol, phase, contract, etc). Relevant patient information is collected from the original DICOM files and EHR files. The collected patient information is saved in a separate Excel file in a de-identified form to maintain a link to related medical images in the same dataset.

Pat denotes de-identified patient ID, Mod denotes modality of image, Phase denotes phase or sequence of image, Cl1, Cl2, Cl3 represents available segmentation classes. IM, LP, and HV denotes the image, liver parenchyma, and hepatic vein respectively.

### 3.2 Results on liver parenchymna and heptasic vessels validation

We conducted a thorough evaluation of our proposed workflow using our data. In Table 1, we present an overview of the MDS structured data (Cl-Class), highlighting its key components. To acquire the training data, specifically focusing on the liver parenchyma and liver vessels, we employed algorithm A2, which automatically extracted the relevant information from the structured directory. It is worth noting that algorithm A2 ensures complete anonymization of the data.

**Table 1:** Structured data overview.

**Pat**	**Mod**	**Phase**	**Cl 1**	**Cl 2**	**Cl 3**	**Data**
H001	CT001	Porto	IM	LP	HV	Yes
H002	CT001	Porto	IM	LP	HV	Yes
H003	CT001	Porto	IM	LP	HV	Yes
H004	CT001	Porto	IM	LP	HV	Yes
H005	CT001	Porto	IM	LP	HV	Yes
H006	CT001	Porto	IM	LP	HV	No
H007	CT001	Porto	IM	LP	HV	Yes

To illustrate the process of requesting data for AI training, we provide an example in Table 2. Subsequently, we extracted a set of 50-volume CT image volumes from the MDS using algorithm A2, which incorporates a unique key for linking each volume to its corresponding data in the structured directory. In Tables 3 and 4, we showcase the data request file and the A2-generated key, respectively. These elements serve as essential references for manual correction of AI inferences.

**Table 2:** Data request format for AI training.

**Pat**	**Mod**	**Phase**	**Cl 1**	**Cl 2**
H001	CT001	Porto	IM	LP
H002	CT001	Porto	IM	LP
H003	CT001	Porto	IM	LP
H004	CT001	Porto	IM	LP
H005	CT001	Porto	IM	LP
H006	CT001	Porto	IM	LP
H007	CT001	Porto	IM	LP

**Table 3:** Data request for annotation.

**Pat**	**Mod**	**Phase**	**Cl 1**
H001	CT001	Porto	IM
H002	CT001	Porto	IM
H003	CT001	Porto	IM
H004	CT001	Porto	IM
H005	CT001	Porto	IM
H006	CT001	Porto	IM
H007	CT001	Porto	IM

**Table 4:** Example of de-identification keys **Ns**.

**Pat**	**Mod**	**Phase**	**Cl 1**	**Ns**
H001	CT001	Porto	IM	Case005
H002	CT001	Porto	IM	Case006
H003	CT001	Porto	IM	Case001
H004	CT001	Porto	IM	Case003
H005	CT001	Porto	IM	Case002
H006	CT001	Porto	IM	Case007
H007	CT001	Porto	IM	Case004

We have used 95 CT image volumes with separate label volumes of liver parenchyma, and liver vein (portal and hepatic vein together). The dataset is divided into 75:20 image volumes with the corresponding segmentations for the training and validation of a 3D Residual UNet deep learning network [48], [49].

The Python3, and monai 0.5.3 and its compatible packages were used for segmentation algorithm. The learning rate 0.001, and Softmax were used to train AI algorithm. We trained the AI algorithm using an Intel(R) Core (TM) i7–7700 CPU 4.20 GHz (8 cores), 64 GB RAM, and NVIDIA Geforce GTX 3090 Ti-PCIE-24 GB of Video RAM.

The ground truth manual segmentation creation for the dataset was performed by a medical doctor (both have over 6-year medical image post-processing experience) with 3D Slicer [50] and a radiologist who has segmentation experience work using MedSeg. In Slicer, several basic and advanced segmentation tools and techniques were used to make the ground truth.

Delineation criteria for the liver parenchyma are the following: Ground truth includes liver tissue as a whole organ including lesions, all liver vessels, and bile ducts adjoined to the liver parenchyma viewed on the axial plane. The vena-cava is segmented on one additional axial slice cranially. For vessel segmentation, we consider up to five branches from the main portal and hepatic veins.

**Fig. 3:**
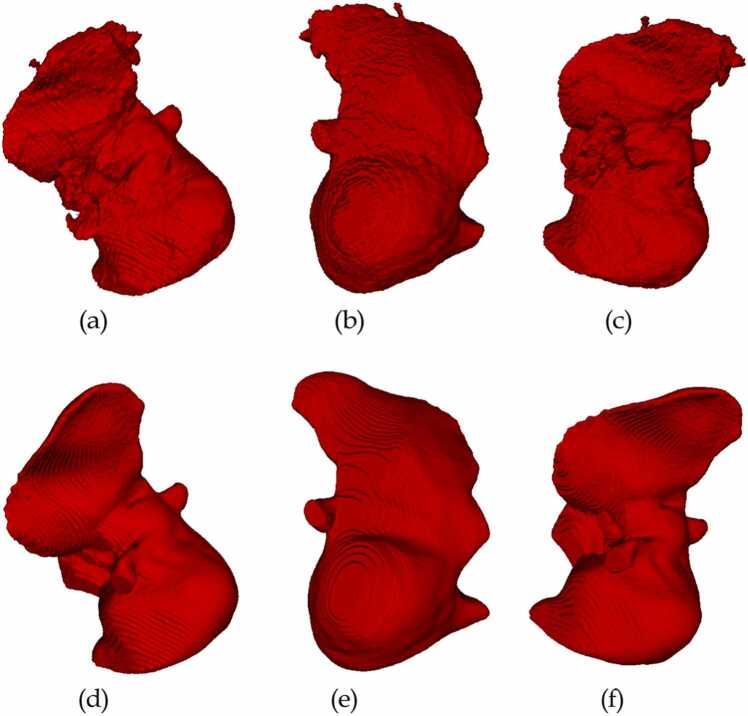
Three different views of liver parenchyma of patient A. (a), (b), (c) are AI predictions. (e), (f), (g) are corresponding manual corrections.

**Fig. 4:**
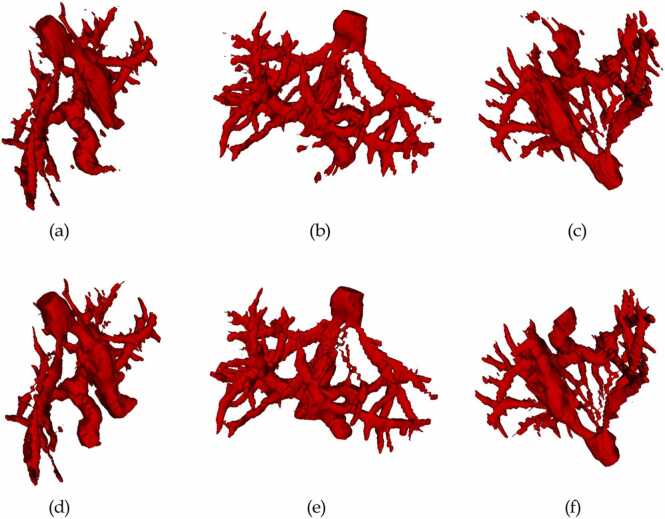
Three different views of liver vessels of randomly selected patient. (a), (b), (c) are AI predictions. (e), (f), (g) are corresponding manual corrections.

We inferred 50 volumes of CT images using the trained deep learning algorithm for liver parenchyma and veins to be used for the evaluation. The differences between AI prediction and manual correction of AI prediction on liver parenchyma and liver vessels are shown in Fig. 3, Fig. 4. The manually corrected labels LP and HV are renamed and moved to the source folder of the MDS directory using the algorithm A3. Then, using Algorithm A1, the labels are moved to the corresponding directories.

We have evaluated DSC, OS, and US scores to measure the accuracy of AI predictions. The obtained metrics for liver parenchyma and hepatic vessels, in averages, are presented in Table 5. It can be observed that the best Dice score is 0. 9821 and the lower Dice score is 0.9366 for liver parenchyma. Also, the best Dice score is 0.9124 and the lower Dice score is 0.4909 for hepatic vessels.

**Table 5:** Quantitative measures of liver parenchyma and vessels using metrics (average scores).

Label	Dice score	Over segmentation	Under segmentation
Liver parenchyma	0.9639	0.0307	0.0412
Hepatic vessels	0.7425	0.2687	0.2462

## 4 Discussions

Studies showed the potential of AI in organising and structuring medical data, but few studies have both automation and manual verification steps. Our workflow provides a practical and adaptable solution.

In this article, AI-assisted ground truth creation is focused on the segmentation of liver parenchyma (LP), liver lesion (LL), portal vein (PV), hepatic vein (HV), liver vein (LV) that includes hepatic and portal vein as a single label class, and bile duct (BD), as shown in Fig. 2. The reason to have individual classes is that images and labels are needed for various retrospective studies in the individual forms of segmentation labels. Combining or fusing different labels of the same image is simply element-wise addition using Python numpy operation.

The manual review and correction process for the liver parenchyma was predominantly efficient, with the majority of corrections being both accurate and requiring only minimal adjustments. This highlights the proficiency of the human reviewers and their ability to effectively work alongside the automated system. In the case of the liver parenchyma, the collaboration between human expertise and artificial intelligence proved to be quite promising.

The vessel segmentation output, although not as reliable, still demonstrated some potential for improvement. While there was a considerable amount of correction needed, this presented a valuable opportunity for refining the AI algorithms and enhancing the overall quality of the vessel segmentation process. In certain instances, starting the vessel segmentation anew was a more efficient approach than attempting to correct the AI output. This flexibility allowed for better optimization of the process, ultimately leading to improved results. By continually refining and iterating upon the AI system, vessel segmentation accuracy can be expected to increase over time, ultimately strengthening the synergistic relationship between human reviewers and artificial intelligence.

In general, the creation of ground truth for clinical use or AI training using semi-automated region growing algorithm and correction is a time-consuming tedious task. To overcome this challenge, we use a deep learning algorithm to create initial labels. We also automated the structuring, anonymizing, and extraction of data.

The benefits of this semi-automatic workflow can streamline and improve patient care, and improve medical research and development. With this semi-automatic process, data handlers can quickly and easily provide data for various research purposes. The outcomes of the research allow clinicians to make more informed decisions about patient care, and understand in depth, resulting in better outcomes for patients. Another advantage of semi-automated medical data extraction is the ability to save time, reduce the need for manual labour, and minimize the risk of errors.

However, our study has several limitations. Our implementation was tested in a specific environment with NIfTI format medical data, and clinical data as JSON type. Its generalizability to other settings and data types needs further investigation. The integration of this workflow into existing hospital systems may present technical and operational challenges that were not addressed in this study.

## 5 Conclusion

In this work, we have proposed a method for structuring medical data and assisting in creating a big dataset using deep-learning model. The discussed methods were implemented using open-source Python packages. This semi-automated process can extract images and infer them efficiently. Once the inferred images were verified and corrected manually, they can be pulled back to the proposed main directory. The semi-automated algorithms could potentially save clinician work on handling data, extracting data for various research, adding annotated labels again to the corresponding directory, etc. The proposed directory structure is not limited to the liver dataset used in this work, it can be extended to any imaging modality depending on the keywords in the file name. It can be intriguing to incorporate a search engine that utilizes minimal keywords to efficiently extract data from structured data.

## Funding statement

The author Varatharajan Nainamalai was supported by the carrier grant award from Helse Sør-Øst, Norway of Dr. Åsmund Avdem Fretland, Oslo University Hospital, Rikshospitalet, Oslo, Norway for "The transplantation revolution: Living donor liver transplant for colorectal metastases" in 2022 under the grant number: 2022082.

## Ethical statement

For the development and testing of the proposed workflow, a dataset was created with data originating from the Oslo laparoscopic versus open liver resection for colorectal liver metastases clinical trial study (OSLO-COMET).

The study was performed in accordance with the ethical standards of the institutional and/or national research committee and with the 1964 Helsinki Declaration and its later amendments or comparable ethical standards (ClinicalTrials.gov: NCT01516710). The project will comply with Norwegian legislation on research ethics and data protection by following the policies given by Oslo University Hospital.

The use of data in the OSLO-COMET trial has been approved by the Norwegian Regional Ethics Committee (REK Helse Sør-Øst nr. 2011/1285). The written informed consent has been obtained from all patients.

## CRediT authorship contribution statement

**Olle Jacob Elle:** Supervision, Resources, Project administration, Investigation, Funding acquisition. **Bjørn Edwin:** Project administration, Investigation, Funding acquisition. **Asmund Avdam Fretland:** Visualization, Supervision, Project administration, Methodology, Investigation, Funding acquisition, Data curation. **Håvard Bjørke Jenssen:** Visualization, Validation, Investigation, Data curation. **Ilanko Balasingam:** Supervision, Project administration, Funding acquisition. **Hemin Ali Qair:** Writing – review & editing, Writing – original draft, Investigation, Conceptualization. **Varatharajan Nainamalai:** Writing – review & editing, Writing – original draft, Visualization, Validation, Software, Resources, Project administration, Methodology, Investigation, Formal analysis, Data curation, Conceptualization. **Egidijus Pelanis:** Writing – review & editing, Writing – original draft, Visualization, Validation, Data curation, Conceptualization.

## Declaration of Competing Interest

The authors declare that they have no known competing financial interests or personal relationships that could have appeared to influence the work reported in this paper.
