# Supplementary material for: Automated algorithm for medical data structuring, and segmentation using artificial intelligence within secured environment for dataset creation
Source: Eur J Radiol Open. 2024 Jun 27;13:100582. doi: 10.1016/j.ejro.2024.100582 (PMC11260947; doi:10.1016/j.ejro.2024.100582)
Supplement: Supplementary file 1 — Supplementary material [file mmc1.pdf]

Algorithm 1 Algorithm for creating, and moving data for different directories

```
Input: CSV input file, NIfTI images
Inputs ← READ files in the input directory
CSV file ← FIND a CSV file in Inputs
if CSV file NOT empty then
    ClinicalData ← READ contents of CSV file
end if
for file in Inputs do
    if file is NOT CSV then
        CREATE list L ← SPLIT file based on underscores
        Level 2, Level 3, Level 4 ← L[0], L[1], L[2]
        if any of Level 2, Level 3, Level 4 directory NOT exists then
            CREATE Level 2, Level 3, Level 4 directories
        end if
        MOVE file TO Level 4 directory
        if L[0] in Clinical Data then
            Patient Data ← Clinical Data L[0]
            Patient Data.json ← CONVERT Patient Data TO JSON format
            SAVE Patient Data.json in Level 2 directory
        end if
    end if
end for
ASSIGN list C ← images, possible labels
CREATE a new CSV file WITH header C
folders ← READ all the folders in Level 2 directory
for folder in folders do
    List F ← empty
    GOTO full-depth of each folder
    SEARCH file with the keyword in C
    if key in C found then
        APPEND key in F
    end if
    SORT the list F as the same order as C
    WRITE the list F in overview.csv
end for
```

Algorithm 2 Data extraction algorithm for various studies and ground-truth creation

```
Input:csv input file, NIfTI images
CREATE an empty list csvrows
for i, rows in enumerate (dataset request csv file) do
    csvrows ← rows
end for
sequence ← shuffle randomly natural numbers N_s
CREATE a CSV file named relation.csv WITH system date
if requested data for ground-truth creation then
    WRITE sequence and Patient id. IN relation.csv
end if
for row in csvrows do
    for data in possible depth of Level 2 folders do
        if request key matches then
            COPY the data of the NIfTI image
            Output: Save NIfTi image with sequence
        end if
    end for
    Patient Data ← READ patient data in a .json file
    json data ← OPEN Patient Data
    REPLACE patient id. WITH sequence in the .json data
    SAVE the new .json data
end for
```

Algorithm 3 Algorithm for moving data from after manual correction

```
Input:csv input file, NIfTI images
PICK the relation.csv WITH the created date
CREATE an empty list rows
for row in relation.csv do
    APPEND row in rows
end for
for image in manually edited images directory do
    Rename sequence with Patient id.
    MOVE images to the source directory of the BIDS dataset
end for
```
